# Depressive and Anxiety Symptoms Predict Health-Related Quality of Life More than Cognitive Impairment After Minor Stroke or Transient Ischemic Attack: A Hierarchical Regression Analysis

**DOI:** 10.3390/healthcare14070948

**Published:** 2026-04-04

**Authors:** María Rocío Córdova-Infantes, José María Ramírez-Moreno

**Affiliations:** 1 Department of Neurology, University Hospital Virgen de Valme, 41014 Seville, Spain; marcorinf@gmail.com; 2 Department of Biomedical Sciences, University of Extremadura, 06006 Badajoz, Spain; 3 Department of Neurology, University Hospital of Badajoz, 06080 Badajoz, Spain

**Keywords:** transient ischemic attack, minor stroke, depression, anxiety, quality of life, hierarchical regression, mediation analysis

## Abstract

**Highlights:**

**What are the main findings?**

- Mood symptoms are the dominant determinants of health-related quality of life after TIA or minor stroke.
- Cognitive impairment has no meaningful independent impact on quality of life once mood is accounted for.

**What are the implications of the main findings?**

- Anxiety and depressive symptoms are highly prevalent after TIA or minor stroke and strongly affect health-related quality of life.
- Cognitive impairment is common but does not independently predict quality of life once mood symptoms are considered.
- Mood and cognitive complications follow separate pathways, highlighting the importance of routine psychological assessment.

**Abstract:**

**Background:** Transient ischemic attack (TIA) and minor stroke often result in excellent functional recovery but are frequently followed by substantial psychological morbidity. It remains unclear whether mood disturbances or cognitive impairment are the primary contributors to reduced health-related quality of life (HRQoL) in this population. **Methods:** We conducted a prospective observational case–control study including 90 patients with acute TIA or minor stroke confirmed by diffusion-weighted imaging and 92 age-matched healthy controls. At 90 days, participants completed the Hamilton Depression Rating Scale, Hamilton Anxiety Rating Scale, Montreal Cognitive Assessment, and the EQ-5D-5L. Hierarchical multiple regression using standardized z-scores identified independent predictors of HRQoL. Bias-corrected bootstrapped mediation analyses (5000 iterations) assessed whether cognitive impairment mediated the relationship between mood symptoms and HRQoL. **Results:** Compared with controls, patients exhibited markedly higher rates of depressive symptoms (82.2% vs. 18.5%), anxiety symptoms (81.1% vs. 21.7%), and cognitive impairment (66.7% vs. 13.0%) (all *p* < 0.001). Psychopathological variables explained an additional 36.6% of HRQoL variance, whereas cognitive and neuroimaging variables contributed only 1.7% (ΔR^2^ = 0.017; *p* = 0.523). In the fully adjusted regression model, HAM-A showed the numerically largest standardized coefficient (β = −0.055; *p* = 0.064), representing a trend toward significance, while HDRS-17 did not individually reach statistical significance (β = −0.043; *p* = 0.147); cognitive impairment had negligible independent effects (β = −0.001; *p* = 0.947). Both mood variables collectively accounted for the substantial majority of explained HRQoL variance, far exceeding the contribution of cognitive and neuroimaging predictors. Mediation analyses revealed no significant indirect effects, indicating that mood and cognitive complications are statistically consistent with a model in which mood and cognitive symptoms exert independent effects on HRQoL; temporal ordering cannot be established from these cross-sectional measures. **Conclusions:** Following TIA or minor stroke, depressive and anxiety symptoms are highly prevalent, persist despite good neurological recovery, and exert a disproportionately negative impact on HRQoL. Anxiety appears particularly influential in determining patient-reported outcomes. The statistical consistency of the mediation models with parallel rather than sequential mood–cognition pathways suggests that these represent independent neurobiological sequelae requiring separate clinical attention, underscoring the need for routine and concurrent assessment of both mood and cognitive function after TIA and minor stroke.

## 1. Introduction

Cerebrovascular disease remains one of the leading global causes of morbidity and disability, with an estimated 11.9 million incident cases each year [1]. Although research has traditionally focused on moderate-to-severe stroke, transient ischemic attacks (TIAs) and minor ischemic strokes represent a substantial proportion of cerebrovascular events, accounting for up to 16% of first-ever cases in population-based cohorts [2]. Historically considered to have minimal long-term consequences due to the absence of significant functional disability (modified Rankin Scale ≤ 2) [3,4], these events are now recognized as potential triggers for persistent psychopathological symptoms and cognitive impairment, even in patients with excellent neurological recovery and no recurrent vascular events [5,6].

Post-stroke depression is among the most prevalent neuropsychiatric complications in this population, affecting 11% to 42% of individuals after TIA or minor stroke depending on study design, timing, and patient characteristics [7,8,9,10]. Anxiety is also common, reported in 12% to 30% of cases, and frequently co-occurs with depressive symptoms in a bidirectional pattern that amplifies emotional distress and functional difficulties [9,11,12]. Importantly, these symptoms can arise even in the absence of detectable physical disability, implicating mechanisms beyond motor impairment, including neurobiological alterations, inflammatory pathways, and psychosocial stressors [13,14].

Cognitive impairment is another frequently under-recognized sequela, reported in approximately 30–54% of TIA and minor stroke survivors [15,16,17,18] often coexisting with depressive and anxiety symptoms. Although cross-sectional studies [15,16] have been conducted, no previous study has directly evaluated whether cognitive impairment causally precedes mood alterations, contributes independently to emotional difficulties, or whether these complications emerge as parallel consequences of cerebrovascular injury.

Mood disturbances have a substantial impact on health-related quality of life (HRQoL), functional independence, return to work, and rehabilitation engagement [17]. In patients with severe stroke, the HRQoL is primarily influenced by motor and functional limitations. However, the mechanisms underlying HRQoL reductions in minor cerebrovascular events, where physical disability is minimal, remain poorly characterized.

Despite evidence linking depression, anxiety, and cognitive impairment to HRQoL after minor stroke, the relative contributions of these factors and potential pathways connecting them remain unclear. Mediation analysis offers a suitable approach to clarify whether (1) cognitive impairment leads to mood disturbances that subsequently reduce HRQoL, (2) cognitive impairment and mood disturbances exert independent effects, or (3) both mechanisms operate concurrently.

It should be emphasised that the mood and cognitive symptoms described in this study are not conceptualised as purely reactive or functional responses to the experience of illness. A substantial and growing body of mechanistic evidence indicates that ischemic injury, even when clinically minor, can produce neurobiological changes sufficient to disrupt mood regulation and cognitive function through direct cortico-limbic circuit disconnection, neuroinflammatory signalling, hypothalamic–pituitary–adrenal axis dysregulation, and impaired neuroplasticity [13,14]. This neurobiological framing has direct implications for how post-stroke psychological and cognitive sequelae should be assessed, interpreted, and treated.

Anxiety appears particularly influential in determining patient-reported outcomes. The statistical consistency of the mediation models with parallel rather than sequential mood–cognition pathways suggests that these represent independent neurobiological sequelae requiring separate clinical attention, underscoring the need for routine and concurrent assessment of both mood and cognitive function after TIA and minor stroke. Additionally, we assessed the utility of neuroimaging biomarkers as predictors of neuropsychiatric and cognitive outcomes in this population.

## 2. Materials and Methods

### 2.1. Study Design and Participants

We conducted a single-center, prospective, observational case–control study. Consecutive patients aged 18–70 years presenting with acute TIA or minor ischemic stroke (NIHSS ≤ 4) and confirmed by magnetic resonance imaging (MRI) with diffusion-weighted imaging (DWI) were enrolled.

Exclusion criteria included prior dementia, significant pre-existing disability (pre-morbid modified Rankin Scale [mRS] > 1), and inability to communicate in Spanish. Controls were age-matched individuals with no history of cerebrovascular events, dementia, or major neurological disease.

Neuroimaging data were obtained exclusively for the case group as part of the standard clinical workup for TIA and minor stroke. Brain MRI was not performed in the control group, as controls were healthy volunteers without neurological indications for imaging. The prevalence of subclinical structural brain abnormalities in the control group is therefore unknown, which precludes direct imaging-based case–control comparisons.

This study adhered to the STROBE reporting guidelines, and the protocol was approved by the Ethics Committee of the University Hospital of Badajoz (Spain). Written informed consent was obtained from all participants.

Demographic characteristics, vascular risk factors, and neurological status—including NIHSS subscores for consciousness, language, and motor function—were collected. The functional status at discharge was assessed using the mRS. Standardized physical measurements (blood pressure, height, weight, and body mass index) were obtained according to institutional protocols. Laboratory studies performed on admission included plasma glucose, urea, creatinine, estimated glomerular filtration rate, total cholesterol, HDL cholesterol, LDL cholesterol, and triglyceride levels.

All psychopathological, cognitive, and HRQoL assessments were conducted by research team members at the University Hospital of Badajoz with specific training in the administration of the study instruments, following a standardised structured protocol developed for this study. Neuroimaging analyses were performed by experienced neuroradiologists at the same centre who were not part of the psychopathological assessment team and were blinded to all clinical and psychological outcome data.

### 2.2. Neuroimaging Acquisition and Analysis

All patients underwent brain MRI in the acute phase (mean 1.3 ± 0.8 days post-event) using a Philips Intera 1.5-Tesla scanner. The imaging protocol was optimised for acute lesion detection and standard white matter burden quantification. It did not include advanced sequences (e.g., arterial spin labelling, diffusion tensor imaging, or high-resolution 3T sequences) capable of detecting microstructural injury, altered perfusion, or network-level disconnection, which may contribute to neuropsychiatric and cognitive symptoms independently of macrostructural lesion burden. The protocol included:

DWI: presence, number, and location of acute ischemic lesions.

Apparent diffusion coefficient (ADC) maps: confirmation of acute ischemia.

FLAIR: quantification of white matter hyperintensities using the Age-Related White Matter Changes (ARWMC) scale.

T2-weighted gradient echo: presence, number, and location of cerebral microbleeds.

Neuroimaging analyses were performed by experienced neuroradiologists who were blinded to all clinical and psychopathological outcomes. Image quality was reviewed systematically and cases with incomplete or non-diagnostic sequences were excluded from imaging-based analyses.

### 2.3. Psychopathological, Cognitive, and HRQoL Assessment

All assessments were conducted 90 ± 14 days after the index event by evaluators blinded to neuroimaging findings.

Depression: Hamilton Depression Rating Scale (HDRS-17); scores ≥ 7 defined depressive symptoms, consistent with the lower boundary of the mild symptom range [18]. This instrument has been validated as a screening tool in post-stroke populations [19].

Anxiety: Hamilton Anxiety Rating Scale (HAM-A) [20]; clinically relevant anxiety symptoms defined as HAM-A ≥ 7, consistent with prior work in post-stroke and TIA populations [21,22].

Montreal Cognitive Assessment (MoCA; 30-point scale); scores < 26 defined cognitive impairment. In accordance with the recommendation of Nasreddine et al. [23], a 1-point correction was added for participants with 12 or fewer years of formal education, so that the applied threshold effectively reflects education-adjusted scoring. The MoCA has been validated in Spanish-speaking patients with TIA/minor stroke, and this education-adjusted cut-off has demonstrated adequate sensitivity and specificity in this specific population [24].

Health-related quality of life (HRQoL): EuroQol EQ-5D-5L utility index and visual analogue scale (EQ-VAS, 0–100) [25]. Utility values were derived using the Spanish value set [26].

Each instrument was applied using its validated Spanish-language version, with specific validation references provided where first introduced above.

To minimize interviewer-related bias, all psychopathological scales were administered following a standardized structured protocol. The prevalence figures reported here represent the proportion of participants meeting screening-based symptom thresholds and should not be interpreted as rates of clinically diagnosed depressive or anxiety disorders.

### 2.4. Statistical Analysis

Continuous variables are expressed as mean ± SD or median [IQR], and categorical variables as numbers (%). Between-group comparisons used independent-samples *t* tests or Mann–Whitney U tests, and χ^2^ or Fisher’s exact tests were used for categorical variables. Effect sizes were expressed as Cohen’s *d* and odds ratios (ORs) with 95% confidence interval (CIs). Statistical significance was set at *p* < 0.05.

Among the cases, bivariate associations were examined using Pearson or Spearman correlation coefficients, depending on the variable distribution and scale. Correlation strength was interpreted as weak (|r| < 0.30), moderate (0.30–0.70), or strong (>0.70). False-discovery-rate–adjusted *q* values are provided in Supplementary Materials.

### 2.5. Regression Modeling

Independent predictors of HRQoL (EQ-5D-5L utility index) were examined using hierarchical multiple linear regression. Three nested models were prespecified as follows:

Sociodemographic/clinical: age, sex, mRS, social risk.

Psychopathological: HDRS-17, HAM-A.

Cognitive/neuroimaging: MoCA, presence of DWI lesions, prior silent infarcts.

Continuous predictors were standardized (z-scores); binary variables were coded 0/1. Model fit was evaluated with R^2^, adjusted R^2^, ΔR^2^, and incremental *F* tests. For each predictor, we report the standardized (β), SE, 95% CI, and *p* values.

Given the bounded nature of EQ-5D-5L utility values and the violation of the normality assumption (Shapiro–Wilk: W = 0.937, *p* = 0.003), beta regression was applied as a pre-specified sensitivity analysis, with utility values transformed to the open unit interval using the formula (y × (n − 1) + 0.5)/n. HC3-robust standard errors were additionally computed. Findings were unchanged across all sensitivity analyses.

### 2.6. Mediation Analysis

Psychological mediation models were evaluated using non-parametric bootstrapped mediation. Two models were tested: (a) MoCA → HDRS-17 → EQ-5D-5L and (b) MoCA → HAM-A → EQ-5D-5L.

All variables were standardized, and age and sex were included as covariates. Indirect effects were considered significant if the 95% bootstrap confidence interval (CI) excluded zero.

Mediation analyses were conducted using PROCESS v3.5 (SPSS v29) with 5000 bias-corrected accelerated bootstrap resamples; all other analyses were performed in R (v4.3.0).

### 2.7. Data Availability

The data supporting the findings of this study are available from the corresponding author upon reasonable request.

## 3. Results

### 3.1. Sample Characteristics

A total of 182 participants were enrolled, including 90 patients with acute TIA or minor ischemic stroke (NIHSS ≤ 4) and 92 age-matched healthy controls. The case group had a significantly higher proportion of males (73.3% vs. 45.7%; χ^2^ = 13.32; *p* < 0.001) and a greater prevalence of hypertension (58.9% vs. 37.0%; *p* = 0.003), diabetes mellitus (28.9% vs. 15.2%; *p* = 0.037), current smoking (63.3% vs. 38.0%; *p* < 0.001), and prior ischemic heart disease (13.3% vs. 1.1%; *p* = 0.004). Among cases, acute DWI lesions were present in 72.2% (n = 65), previous silent infarcts in 28.9% (n = 26), and cerebral microbleeds in 7.8% (n = 7). The baseline characteristics are summarized in Table 1, with extended imaging descriptors in Table S1 (baseline block).

These baseline differences in sex distribution and vascular risk factor burden are clinically relevant, as each of these factors is independently associated with mood, cognitive function, and health-related quality of life. Between-group differences in psychopathological, cognitive, and HRQoL outcomes should therefore be interpreted with caution, as they may partly reflect pre-existing group differences rather than the cerebrovascular event per se. Notably, the primary regression analyses—which constitute the main analytical contribution of this study—were conducted exclusively within the case group and are not affected by the composition of the control group.

### 3.2. Psychopathological, Cognitive, and HRQoL Outcomes at 90 Days

**Figure 1:**
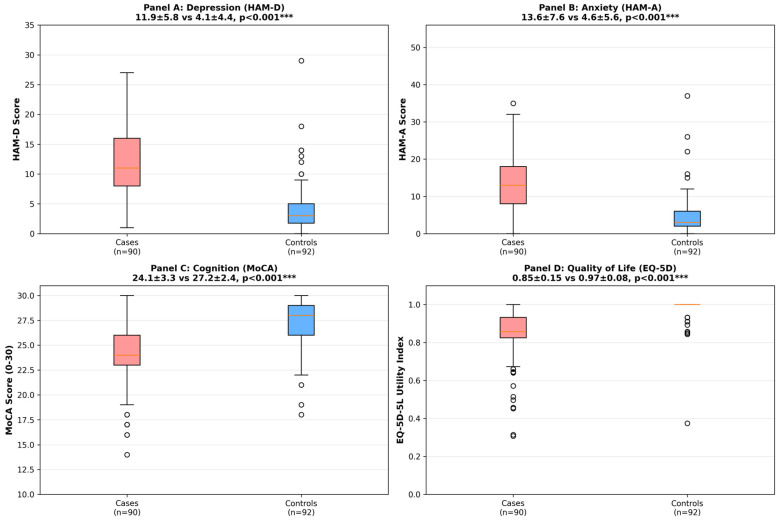
Comparison of Depressive Symptoms, Anxiety, Cognition, and Quality of Life Between TIA/Minor Stroke Patients and Healthy Controls. Comparison of depressive symptoms (HDRS-17), anxiety (HAM-A), cognitive performance (MoCA), and quality of life (EQ-5D-5L) between TIA/minor stroke patients (n = 90) and healthy controls (n = 92). Boxes represent the median and interquartile range; whiskers indicate minimum and maximum values, excluding outliers. Group comparisons were performed using independent-samples tests as appropriate. *** *p* < 0.001.

Depressive (HDRS 17 ≥ 7) and anxiety symptoms (HAM-A ≥ 7) were significantly more prevalent in cases than controls (depression: 82.2% vs. 18.5%; χ^2^ = 71.42; *p* < 0.001; anxiety: 81.1% vs. 21.7%; χ^2^ = 61.82; *p* < 0.001). Patients also showed higher mean symptom scores (HDRS 17: 11.86 ± 5.84 vs. 4.13 ± 4.35, *p* < 0.001, d = 1.50; HAM-A: 13.60 ± 7.57 vs. 4.64 ± 5.58, *p* < 0.001, d = 1.35). Cognitive impairment (MoCA < 26) was more frequent among cases (66.7% vs. 13.0%; χ^2^ = 52.49; *p* < 0.001), with lower mean MoCA scores (24.08 ± 3.26 vs. 27.21 ± 2.36; *p* < 0.001, d = −1.10). HRQoL (EQ 5D 5L utility) was markedly reduced in cases (0.847 ± 0.152) compared with controls (0.974 ± 0.076; *p* < 0.001, d = −1.05). These contrasts are summarized in Figure 1 and, in greater detail (including Cohen’s d, ORs, and 95% CIs), in Table S1. The case–control prevalences are shown in Figure S1A.

### 3.3. Bivariate Associations

Within cases, mood scores showed strong negative associations with HRQoL (HDRS 17 vs. EQ 5D 5L r = −0.612, *p* < 0.001; HAM-A vs. EQ 5D 5L r = −0.625, *p* < 0.001), and HDRS 17 correlated strongly with HAM-A (r = 0.681, *p* < 0.001), whereas correlations between MoCA and HRQoL were negligible (r = 0.092, *p* = 0.372). The complete correlation matrix (including correlation type and FDR adjusted q values) is provided in Table S2 and is graphically displayed in Figure S1B.

### 3.4. Predictors of HRQoL: Hierarchical Regression

**Figure 2:**
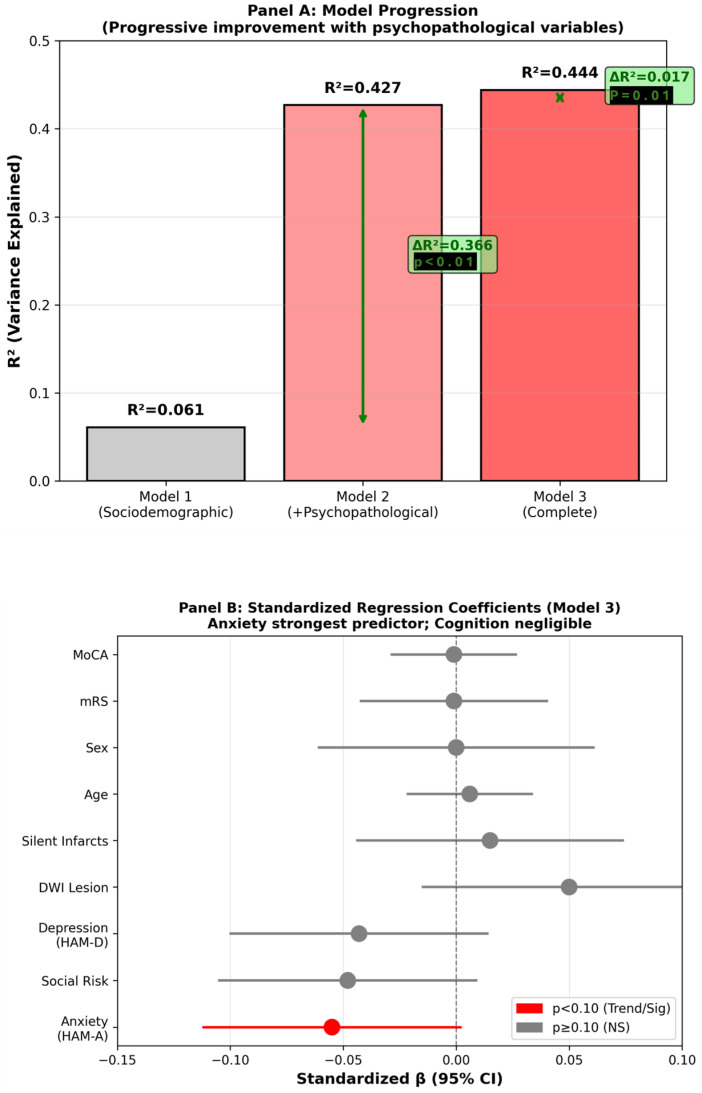
Hierarchical Regression Analysis: Model Progression and Predictor Contribution. Hierarchical regression model performance and standardized coefficients. (**A**) shows the variance explained (R^2^) for each incremental model and the corresponding change in explained variance (ΔR^2^). (**B**) displays the standardized regression coefficients (β) and 95% confidence intervals for the predictors in the final model. Red markers denote trend-level effects (0.05 ≤ *p* < 0.10). HAM-A: Hamilton Anxiety Rating Scale; HAM-D: Hamilton Depression Rating Scale; mRS: modified Rankin Scale; MoCA: Montreal Cognitive Assessment; DWI: diffusion-weighted imaging; NS: not significant.

Among cases with complete data (n = 89), Model 1 (age, sex, mRS and social risk) accounted for 6.1% of the HRQoL variance (F = 1.36; *p* = 0.256). Adding depressive and anxiety symptoms to Model 2 significantly increased the explained variance (ΔR^2^ = 0.366; total R^2^ = 0.427; F = 10.18; *p* < 0.001). Model 3, which added MoCA, DWI lesion status, and prior silent infarcts, contributed minimally (ΔR^2^ = 0.017; R^2^ = 0.444; F = 7.02; *p* < 0.001); the increment over Model 2 was not significant (ΔR^2^ = 0.017; *p* = 0.523). Model-level metrics are presented in Table 2; full coefficients for Model 3 (β, SE, 95% CI, *p*) in Table 3. Model assumptions and robustness were verified through standard diagnostic and sensitivity analyses, which did not materially alter the results. Figure 2 illustrates the stepwise increase in R^2^ (A) and the standardized coefficients for the final model (B).

### 3.5. Mediation Analysis

In the depression model (Model A), MoCA was not associated with HRQoL (β = 0.056; *p* = 0.609) or depression severity (a path β = −0.095; *p* = 0.381). Depression was associated with lower HRQoL (b path β = −0.060; *p* = 0.034). The indirect effect was small and nonsignificant (β = −0.006; 95% BCa CI −0.068 to 0.047). Similarly, in the anxiety model (Model B), MoCA was not associated with anxiety (a path β = −0.127; *p* = 0.239), whereas anxiety predicted lower HRQoL (b path β = −0.062; *p* = 0.041). The indirect effect was nonsignificant (β = 0.008; 95% BCa CI −0.046 to 0.078).

**Figure 3:**
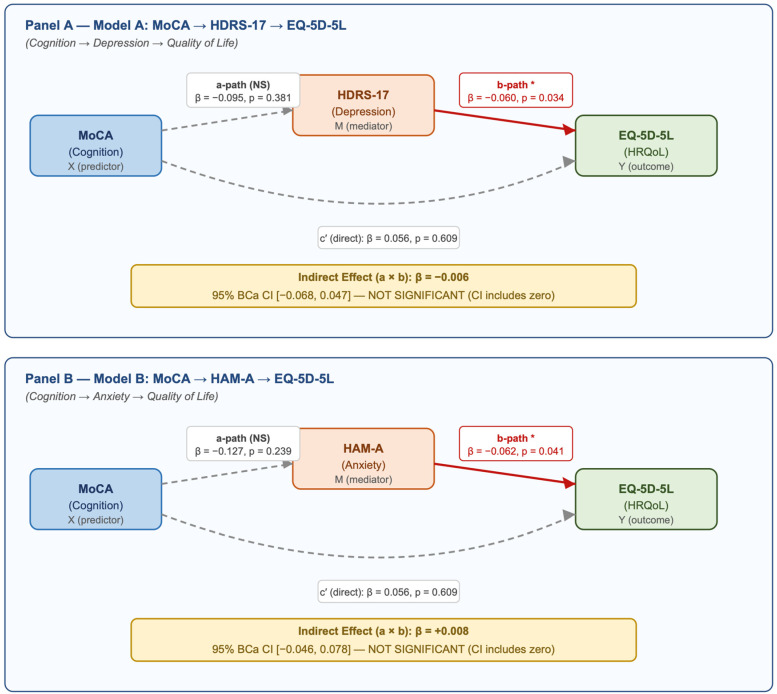
Mediation Analysis: Indirect Effects of Depression and Anxiety on the Cognition–Health-Related Quality of Life Relationship. Legend: Mediation path diagrams for Model A (MoCA → HDRS-17 → EQ-5D-5L) and Model B (MoCA → HAM-A → EQ-5D-5L). Standardised coefficients (β) and *p*-values are shown on each path. Solid red arrows indicate significant paths (*p* < 0.05); dashed grey arrows indicate non-significant paths. Shaded boxes show the indirect effect (a × b) with bias-corrected accelerated (BCa) bootstrap 95% confidence intervals (5000 resamples). All variables are standardised (z-scored); age and sex covariates are included in all models. * *p* < 0.05.

Path level estimates (c, a, b, c′) and bootstrap intervals are detailed in Table S3A,B; and the standardized path diagrams are shown in Figure 3.

### 3.6. Sex-Stratified Analysis

Sex specific means for HDRS 17, HAM-A, MoCA, and EQ 5D 5L are presented in Figure S1, Panel C; statistical tests and effect sizes are reported in Table S1 (sex-stratified block). This analysis complements the primary models and allows assessment of potential sex-related differences in clinical profiles.

## 4. Discussion

### 4.1. Neurobiological Framing of Post-Stroke Mood and Cognitive Symptoms

The mood and cognitive symptoms documented in this study should not be interpreted as primarily ‘psychological’ reactions to the experience of illness. A substantial body of mechanistic evidence indicates that ischemic lesions, even those considered ‘minor’, can produce neurobiological changes sufficient to disrupt mood regulation and cognitive function through direct cortico-limbic circuit disconnection, neuroinflammatory signalling, hypothalamic–pituitary–adrenal axis dysregulation, and impaired neuroplasticity [13,14,27]. The fact that mood symptoms were not significantly associated with structural lesion burden in our models does not exclude biological causation; it may instead reflect the limited sensitivity of conventional structural MRI for microstructural injury, perfusion abnormalities, and network-level disconnection that may underpin symptom generation.

### 4.2. Prevalence and Clinical Significance of Psychopathological and Cognitive Complications

This study highlights a notable paradox in outcomes following TIA and minor ischemic stroke. Despite excellent functional recovery—with nearly 60% of patients achieving mRS = 0—a substantial burden of psychopathological and cognitive complications persists [4,26]. We observed high rates of depressive symptoms (82.2%), anxiety symptoms (81.1%), and cognitive impairment (66.7%), far exceeding those in age-matched healthy controls (18.5%, 21.7%, and 13.0%, respectively), with effect sizes ranging from 1.10 to 1.50. These findings challenge the traditional labelling of TIA and minor stroke as benign events.

The prevalence figures align with studies using symptom-based thresholds rather than diagnostic criteria, which systematically yield higher estimates than structured diagnostic interviews [28]. Contemporary evidence confirms that stroke survivors have nearly threefold higher odds of depression than the general population, reinforcing that elevated symptom rates reflect genuine neuropsychiatric morbidity rather than measurement artefact [29]. Depressive symptom prevalence in our cohort exceeds that reported in studies using comparable screening thresholds (~60%) [30] and is substantially higher than estimates from studies relying on clinical diagnosis (11–41%) [7,31,32], likely reflecting methodological differences in outcome definition rather than true population differences. The mean HDRS-17 score of 11.86 ± 5.84 in cases, whilst in the mild range, was associated with a large effect size (Cohen’s d = 1.50), supporting clinical relevance beyond statistical significance.

The 81.1% prevalence of anxiety symptoms represents a burden that has been infrequently quantified in TIA and minor stroke populations, where reported rates range from 20% to 55% [9,12,33]. Anxiety has historically been under-investigated relative to depression and cognitive outcomes in this population, and our findings underscore the need for its routine assessment.

Cognitive impairment (MoCA < 26) was detected in 66.7% of cases, at the upper end of the range reported for minor stroke and TIA populations (30–67%) [6,24]. Critically, the high prevalence of cognitive impairment despite excellent motor recovery (mRS ≤ 2 in all patients, mRS = 0 in ~60%) highlights a fundamental limitation of conventional neurological outcome measures: the mRS does not assess cognitive function and therefore systematically underestimates the burden of incomplete neurological recovery. Good functional outcome as defined by disability scales should not be equated with complete neurological recovery, and cognitive assessment should be incorporated as a routine component of post-TIA and post-minor-stroke follow-up, independent of functional disability status. These findings are consistent with recent evidence that lasting impairments—including mood disturbance, fatigue, and cognitive change—persist after TIA and minor stroke and remain underrecognised and inconsistently treated [34].

### 4.3. Mood Symptoms as the Primary Determinants of HRQoL

A key finding of this study is the predominant contribution of psychopathological symptoms to HRQoL, accounting for 36.6% of incremental variance (Model 2 ΔR^2^ = 0.366; *p* < 0.001), compared with 1.7% explained by cognitive and neuroimaging variables (Model 3 ΔR^2^ = 0.017; *p* = 0.523). Once psychopathological variables were included, cognitive impairment and neuroimaging markers showed no significant additional association with HRQoL. These findings indicate that, in this patient population, mood symptom burden is more strongly associated with patient-reported health status than traditional neurological or imaging-based measures.

HAM-A showed the numerically largest standardised coefficient in the fully adjusted model (β = −0.055; *p* = 0.064), representing a trend-level association, while HDRS-17 did not individually reach conventional significance (β = −0.043; *p* = 0.147). This pattern reflects the high collinearity between the two mood scales (r = 0.681; *p* < 0.001), which distributes shared variance across both predictors and attenuates individual coefficients. The collective ΔR^2^ of 0.366 attributable to the psychopathological block therefore provides a more reliable estimate of the mood–HRQoL association than the coefficient of either scale alone. The key finding is the collective and substantial contribution of both mood variables to HRQoL variance, rather than the superiority of one over the other [35].

### 4.4. Independence of Mood and Cognitive Pathways

The mediation models yielded results statistically consistent with anxiety and depressive symptoms each contributing independently to reduced HRQoL, rather than operating through sequential mechanisms, extending previous work on post-stroke psychopathology. The lack of significant indirect effects indicates that cognitive dysfunction represents a parallel neurobiological consequence of cerebrovascular injury rather than a mediator of mood-related HRQoL reduction. These findings are compatible with recent longitudinal data showing that a first-ever DWI-negative TIA is associated with subsequent cognitive decline independent of vascular and demographic factors, implying that cognitive and affective trajectories may be at least partially dissociable [16]. Each domain therefore warrants independent monitoring and targeted intervention [36].

It is important to note, however, that because all neuropsychiatric and quality-of-life measures were obtained concurrently at the 90-day follow-up visit, the mediation models can only demonstrate statistical consistency with a parallel-pathway hypothesis; they cannot establish the temporal ordering of these associations. Prospective longitudinal designs with repeated assessments are required to test causal sequencing and to distinguish antecedent from consequent variables [37].

### 4.5. Neurobiological Mechanisms

Multiple neurobiological mechanisms have been implicated in the genesis of post-stroke mood and cognitive symptoms. Ischemia-related neuroinflammation—characterised by elevated proinflammatory cytokines including IL-6, TNF-α, and CRP—represents a shared biological pathway for both depressive and anxiety symptomatology, operating independently of lesion volume or clinical severity [13,14]. Cerebral small-vessel disease contributes to white matter network disruption and cortico–subcortical disconnection even in the absence of cortical lesions detectable on standard MRI, providing a plausible substrate for mood dysregulation and cognitive impairment after otherwise minor cerebrovascular events. Dysregulation of the hypothalamic–pituitary–adrenal axis and autonomic nervous system dysfunction represent additional pathways linking ischemic injury to somatic anxiety symptoms and altered affective processing [38]. Persistent low-grade neuroinflammation may constitute a treatable biological target independent of formal psychiatric diagnosis, with implications for pharmacological and rehabilitative intervention strategies. Together, these mechanisms support a framework in which anxiety, depressive symptoms, and cognitive impairment are viewed as parallel neurobiological sequelae of cerebrovascular injury rather than reactive psychological responses.

It should furthermore be acknowledged that elevated scores on observer-rated scales such as the HDRS-17 and HAM-A following cerebrovascular events may not exclusively reflect classical primary psychiatric syndromes. Post-stroke mood symptoms may encompass neurologically mediated phenomena—including emotional lability, apathy, irritability, and altered affective processing arising from cortico–subcortical circuit disruption—irrespective of the patient’s subjective emotional state. Distinguishing these neurologically driven presentations from primary psychiatric disorders has direct therapeutic implications: the former may be more responsive to neurobiological interventions, whereas the latter may respond preferentially to psychological therapies. The instruments used in the present study do not allow this distinction to be made, which represents a limitation that future studies incorporating structured diagnostic interviews and neurobiological markers should specifically address.

### 4.6. Neuroimaging Findings and Unexplained Variance

Although DWI lesions were present in 72.2% of cases and prior silent infarcts in 28.9%, these structural findings contributed minimally to HRQoL outcomes, consistent with the hypothesis that neurobiological mechanisms beyond macrostructural lesion burden drive symptom generation. The final model explained 44.4% of HRQoL variance; the remaining 56% is likely attributable to unmeasured predictors including post-stroke fatigue, sleep disturbance, pain, social support quality, return to occupational role, pre-existing personality and coping style, and residual functional limitations below the mRS detection threshold [33,39]. Microstructural brain injury not captured by conventional MRI sequences may also contribute independently to symptom burden [40]. These observations reinforce the need for more comprehensive multidimensional outcome models in future studies.

### 4.7. Empirical Findings and Clinical Implications

It is important to distinguish between what the present data demonstrate empirically and what they suggest clinically. Empirically, this study documents that depressive and anxiety symptoms assessed at 90 days are more strongly associated with self-reported HRQoL than cognitive or neuroimaging markers, and that the mediation models are statistically consistent with mood and cognitive complications arising as parallel, independent neurobiological sequelae. Clinically, this association—if replicated in longitudinal studies and confirmed to be causally modifiable—would support the routine integration of neuropsychiatric assessment into post-stroke follow-up pathways. The present cross-sectional design supports the former but does not by itself establish the latter.

The high prevalence and clinical impact of mood and cognitive symptoms following TIA and minor stroke support the integration of structured neuropsychiatric assessment into post-stroke follow-up pathways [41]. Crucially, these findings should not be interpreted as evidence that the sequelae of cerebrovascular events are primarily or exclusively psychological in nature. Mood symptoms in this context likely reflect a neurobiological continuum ranging from direct ischemic circuit disruption and neuroinflammatory changes to reactive emotional responses to illness. Optimal clinical management therefore requires a multidisciplinary approach encompassing neurological assessment for ongoing or progressive vascular pathology, neurobiological treatment options including pharmacotherapy where indicated and psychological support as a complementary rather than exclusive intervention [42]. Framing post-stroke mood symptoms as mental health problems to be managed by psychological services alone risks deflecting attention from the biological substrate and delaying appropriate neurological re-evaluation.

Sensitivity analyses using beta regression, applied to account for the bounded and non-normally distributed EQ-5D-5L outcome (Shapiro–Wilk: W = 0.937, *p* = 0.003), yielded substantively identical results: psychopathological variables remained the dominant predictors of HRQoL and MoCA contributed no significant incremental predictive value (Wald test *p* = 0.961), confirming the robustness of the primary findings to distributional assumptions about the outcome variable.

### 4.8. Limitations

Several important limitations warrant consideration. First, the concurrent measurement of all neuropsychiatric and quality-of-life variables at a single 90-day time point precludes causal inference; the mediation models demonstrate statistical consistency with a parallel-pathway hypothesis but cannot establish temporal ordering. Prospective longitudinal designs with repeated assessments are required to test causal sequencing and to characterise the longer-term evolution of mood and cognitive trajectories. Second, mood symptoms were quantified using observer-rated screening instruments rather than structured diagnostic interviews; reported prevalence figures therefore reflect symptom-threshold burden rather than clinically diagnosed disorders, and elevated scores may partly represent neurologically mediated phenomena—emotional lability, apathy, circuit-level dysregulation—rather than primary psychiatric syndromes. Pre-event psychiatric history, prior depressive or anxiety diagnoses, and psychotropic medication use were not systematically assessed; it is therefore not possible to determine what proportion of the mood symptoms identified at 90 days represents de novo post-stroke neuropsychiatric sequelae versus the continuation or worsening of pre-existing conditions. Future prospective studies should include admission-phase mood screening using validated instruments such as the PHQ-9 or GAD-7 to allow incident versus prevalent case distinction. Third, although controls were age-matched, the groups differed substantially in sex distribution and vascular risk factor burden, which may confound between-group contrasts in psychological and cognitive outcomes; notably, this does not affect the primary regression analyses, which were conducted exclusively within the case group. Additionally, the absence of cerebrovascular history in controls relied on self-report; given the well-documented underdiagnosis of TIA, subclinical ischemic events in some controls cannot be excluded, which would tend to attenuate the observed between-group differences. Furthermore, neuroimaging data were available exclusively for the case group; structural brain pathology cannot therefore be excluded as a contributor to the psychological and cognitive outcomes observed in controls. Fourth, the 1.5T neuroimaging protocol has limited sensitivity for microstructural injury, cortical microinfarcts, and perfusion abnormalities that may underpin mood and cognitive symptoms independently of macrostructural lesion burden; furthermore, because neuroimaging was performed acutely (mean 1.3 ± 0.8 days post-event), silent interval ischemic events occurring before the 90-day assessment cannot be excluded. The apparent absence of strong imaging–HRQoL associations should therefore not be interpreted as evidence that biological brain injury is unrelated to the observed symptom burden. Finally, the model explained 44.4% of HRQoL variance; unmeasured predictors—particularly post-stroke fatigue, sleep disturbance, and social support—likely account for a substantial proportion of the residual variance. Single-centre recruitment and a predominantly male sample may limit generalisability to broader community populations.

## 5. Conclusions

This study demonstrates that TIA and minor ischemic stroke (NIHSS ≤ 4) are associated with a substantial burden of depressive symptoms, anxiety symptoms, and cognitive impairment that persists despite excellent functional recovery and is strongly linked to reduced HRQoL. Mood symptom burden, rather than cognitive impairment or structural neuroimaging findings, was the primary determinant of patient-reported health status, collectively accounting for 36.6% of incremental HRQoL variance against 1.7% attributable to cognitive and neuroimaging variables.

Critically, the mediation models were statistically consistent with mood and cognitive complications arising as parallel, independent neurobiological sequelae of cerebrovascular injury rather than through a sequential causal chain in which cognitive impairment drives emotional disturbance. This distinction challenges paradigms that focus primarily on motor and functional recovery when interpreting post-event outcomes and has direct implications for how post-stroke follow-up should be structured: each domain requires concurrent and independent assessment and targeted management.

These findings further highlight a fundamental limitation of conventional neurological outcome measures—the mRS does not capture cognitive sequelae, and good functional outcome should not be equated with complete neurological recovery. Given the neurobiological substrate of both mood and cognitive symptoms, optimal post-event care requires a multidisciplinary approach integrating neurological re-evaluation for ongoing vascular pathology, neurobiological treatment options where indicated, and psychological support as a complementary rather than exclusive intervention.

## Figures and Tables

**Table 1:** Baseline demographic characteristics, cardiovascular risk factors, and family history of cases and controls.

Variable	Cases (n = 90)	Controls (n = 92)	*p* Value
**Demographic characteristics**			
Male sex, n (%)	66 (73.3)	42 (45.7)	<0.001
Age, years, mean ± SD	59.3 ± 8.2	58.6 ± 7.8	0.562
Urban residence, n (%)	43 (47.8)	57 (62.0)	0.055
Educational level, n (%)			0.005
─ Literate without formal education	37 (41.1)	20 (21.7)	
─ Primary or secondary education	50 (55.6)	61 (66.3)	
─ University education	3 (3.3)	11 (12.0)	
Social situation, n (%)			0.037
─ Good or acceptable	59 (65.6)	73 (79.3)	
─ Social risk	31 (34.4)	19 (20.7)	
**Cardiovascular risk factors**			
Hypertension, n (%)	52 (57.8)	33 (35.9)	0.003
Dyslipidemia, n (%)	38 (42.2)	29 (31.5)	0.135
Diabetes mellitus, n (%)	26 (28.9)	15 (16.3)	0.042
Atrial fibrillation, n (%)	10 (11.1)	8 (8.7)	0.585
Prior ischemic heart disease, n (%)	11 (12.2)	1 (1.1)	0.002
Current tobacco use, n (%)	57 (64.0)	35 (38.0)	<0.001
Alcohol consumption, n (%)	50 (56.2)	39 (42.4)	0.064
**Family history**			
History of stroke, n (%)	25 (27.8)	27 (29.3)	0.815
History of ischemic heart disease, n (%)	29 (32.2)	30 (32.6)	0.956

Values are presented as mean ± standard deviation or number (percentage). *p* values were calculated using Student’s *t* test for continuous variables and the χ^2^ test or Fisher’s exact test for categorical variables, as appropriate. Statistical significance was set at *p* < 0.001.

**Table 2:** Hierarchical Regression Model Comparison: Incremental Variance Explained in Quality-of-Life Prediction.

Model	Predictors Included	R^2^	Adjusted R^2^	*F* Statistic	*p* Value	ΔR^2^
Model 1	Age, sex, mRS, social risk	0.061	0.016	1.36	0.256	—
Model 2	Model 1 + HDRS-17, HAM-A	0.427	0.385	10.18	<0.001	0.366
Model 3	Model 2 + MoCA, DWI lesion burden, number of infarcts	0.444	0.381	7.02	<0.001	0.017

R^2^ indicates the proportion of variance that is explained by the model. ΔR^2^ represents the incremental change in the explained variance relative to the previous model. mRS: modified Rankin Scale; HDRS-17: Hamilton Depression Rating Scale; HAM-A: Hamilton Anxiety Rating Scale; MoCA: Montreal Cognitive Assessment; DWI: diffusion-weighted imaging.

**Table 3:** Standardized Regression Coefficients for Predictors of Quality of Life.

Predictor	Standardized β	SE	95% CI	*p* Value
Anxiety (HAM-A)	−0.055	0.029	−0.114 to 0.003	0.064 †
Social risk	−0.048	0.029	−0.105 to 0.009	0.100
Depression (HDRS-17)	−0.043	0.029	−0.102 to 0.016	0.147
DWI lesion burden	0.050	0.033	−0.016 to 0.116	0.133
Silent infarcts	0.015	0.030	−0.046 to 0.075	0.627
Age	0.006	0.014	−0.022 to 0.033	0.686
Sex	−0.000	0.031	−0.062 to 0.061	0.990
mRS	−0.001	0.021	−0.044 to 0.041	0.947
MoCA	−0.001	0.014	−0.029 to 0.027	0.947

The values represent the standardized regression coefficients (β). 95% CI indicates the 95% confidence interval. † Trend toward statistical significance (*p* < 0.10). HAM-A: Hamilton Anxiety Rating Scale; HDRS-17: Hamilton Depression Rating Scale; mRS: modified Rankin Scale; MoCA: Montreal Cognitive Assessment; DWI: diffusion-weighted imaging.

## Data Availability

The data that support the findings of this study are available from the corresponding author upon reasonable request.

## Supplementary Materials

The following supporting information can be downloaded at https://www.mdpi.com/article/10.3390/healthcare14070948/s1, Figure S1. Prevalence, correlations, and sex-stratified outcomes at 90 days after TIA/minor stroke. Table S1. Comparison of psychopathological, cognitive, and quality-of-life outcomes (cases vs. controls). Table S2. Correlation matrix among cases (n = 90). Table S3A. Mediation Analysis: Cognitive Function → Depression → Quality of Life (HRQoL). Table S3B. Mediation Analysis: Cognitive Function → Anxiety → Quality of Life (HRQoL). STROBE Checklist—Case–Control Study (Completed).

## Abbreviations

The following abbreviations are used in this manuscript:

TIA	Transient ischemic attack
mRS	Modified Rankin Scale
HRQoL	Health-related quality of life
DWI	Diffusion-weighted imaging
NIHSS	National Institutes of Health Stroke Scale
HDRS-17	Hamilton Depression Rating Scale
HAM-A	Hamilton Anxiety Rating Scale
MoCA	Montreal Cognitive Assessment
