# Supplementary material for: Depressive and Anxiety Symptoms Predict Health-Related Quality of Life More than Cognitive Impairment After Minor Stroke or Transient Ischemic Attack: A Hierarchical Regression Analysis
Source: Healthcare (Basel). 2026 Apr 4;14(7):948. doi: 10.3390/healthcare14070948 (PMC13073151; doi:10.3390/healthcare14070948)
Supplement: Supplementary file 1 [file healthcare-14-00948-s001.zip › healthcare-4200804-supplementary.pdf]

Supplementary Materials

Supplementary A

Table S1. Comparison of psychopathological, cognitive, and quality-of-life outcomes (cases vs controls)

Outcome measure	Cases (n=90)	Controls (n=92)	*p* value	Cohen's *d*	OR (95% CI)†
**DEPRESSION SYMPTOMS**					
HDRS-17, mean ± SD	11.9 ± 5.8	4.1 ± 4.4	<0.001***	1.50	—
Depressive symptoms (HDRS-17 ≥ 7), n (%)	74 (82.2)	17 (18.5)	<0.001***	—	20.41 (9.60–43.42)
**ANXIETY SYMPTOMS**					
HAM-A, mean ± SD	13.6 ± 7.6	4.6 ± 5.6	<0.001***	1.35	—
Anxiety symptoms (HAM-A ≥ 7), n (%)	73 (81.1)	20 (21.7)	<0.001***	—	15.46 (7.50–31.87)
**COGNITIVE FUNCTION**					
MoCA, mean ± SD	24.1 ± 3.3	27.2 ± 2.4	<0.001***	−1.10	—
Cognitive impairment (MoCA < 26), n (%)	60 (66.7)	12 (13.0)	<0.001***	—	13.33 (6.31–28.21)
**QUALITY OF LIFE**					
EQ-5D-5L utility index, mean ± SD	0.85 ± 0.15	0.97 ± 0.08	<0.001***	−1.06	—
EQ-VAS, mean ± SD	51 ± 17	79 ± 13	<0.001***	−1.94	—

Abbreviations: HDRS-17=Hamilton Depression Rating Scale (17-item); HAM-A=Hamilton Anxiety Rating Scale; MoCA=Montreal Cognitive Assessment; EQ-VAS=EuroQol Visual Analogue Scale. ***p*<0.001 (two-sided). Utilities derived using the Spanish EQ-5D-5L value set.

Table S2: Correlation matrix among cases (n=90)

Variable pair	r (type)	p value	q (FDR)	Strength
MoCA ↔ HDRS-17	−0.146 (Pearson)	0.162	0.216	Weak / NS
MoCA ↔ HAM-A	−0.170 (Pearson)	0.112	0.186	Weak / NS
HDRS-17 ↔ HAM-A	0.681 (Pearson)	<0.001***	<0.001	Strong
HDRS-17 ↔ EQ-5D-5L	−0.612 (Spearman)	<0.001***	<0.001	Strong
HAM-A ↔ EQ-5D-5L	−0.625 (Spearman)	<0.001***	<0.001	Strong
MoCA ↔ EQ-5D-5L	0.092 (Spearman)	0.372	0.372	Negligible
Age ↔ HDRS-17	−0.103 (Pearson)	0.314	0.357	Weak / NS
Age ↔ MoCA	−0.081 (Pearson)	0.445	0.445	Weak / NS
DWI lesion (0/1) ↔ HDRS-17	0.137 (Point-biserial)	0.190	0.216	Weak / NS
DWI lesion (0/1) ↔ MoCA	−0.191 (Point-biserial)	0.078	0.117	Weak / trend

Correlation method chosen by variable scale/distribution (Pearson for approximately continuous/normal, Spearman for ordinal/non-normal, point-biserial for binary–continuous). False discovery rate (FDR) controlled within this table using Benjamini–Hochberg. HDRS-17=Hamilton Depression Rating Scale (17-item); HAM-A=Hamilton Anxiety Rating Scale; MoCA=Montreal Cognitive Assessment; EQ-5D-5L=EuroQol five-dimension five-level index; DWI=diffusion-weighted imaging; NS=non-significant.

Table S3A. Mediation Analysis: Cognitive Function → Depression → Quality of Life (HRQoL)

Pathway	Coefficient (β)	SE	95% CI	p value
**c (Total effect)**	0.0560	0.108	[−0.123, 0.235]	0.609
**a (Cognition → Depression)**	−0.0951	0.108	[−0.310, 0.120]	0.381
**c′ (Direct effect)**	0.0572	0.109	[−0.130, 0.245]	0.602
**b (Depression → HRQoL)**	−0.0604	0.028	[−0.116, −0.005]	0.034*
**a×b (Indirect effect)**	−0.0057	—	[−0.068, 0.047]	—

Notes: Standardized (z-scored) variables; covariates: age and sex. Indirect effect estimated via bias-corrected accelerated (BCa) bootstrap with 5,000 resamples. Analyses run with PROCESS v3.5 (SPSS v29). Two-sided tests; $p<0.05$ marked with *. NS = non-significant.

Table S3B. Mediation Analysis: Cognitive Function → Anxiety → Quality of Life (HRQoL)

Pathway	Coefficient (β)	SE	95% CI	p value
**c (Total effect)**	0.0560	0.108	[−0.123, 0.235]	0.609
**a (Cognition → Anxiety Symptoms)**	−0.1272	0.107	[−0.343, 0.088]	0.239
**c′ (Direct effect)**	0.0787	0.110	[−0.145, 0.303]	0.476
**b (Anxiety Symptoms→ HRQoL)**	−0.0620	0.030	[−0.121, −0.003]	0.041*
**a×b (Indirect effect)**	0.0079	—	[−0.046, 0.078]	—

Notes: Standardized (z-scored) variables; covariates: age and sex. Indirect effect estimated via bias-corrected accelerated (BCa) bootstrap with 5,000 resamples. Analyses run with PROCESS v3.5 (SPSS v29). Two-sided tests; $p<0.05$ marked with *. NS = non-significant.

Supplementary B

Figure S1. Prevalence, correlations, and sex-stratified outcomes at 90 days after TIA/minor stroke.

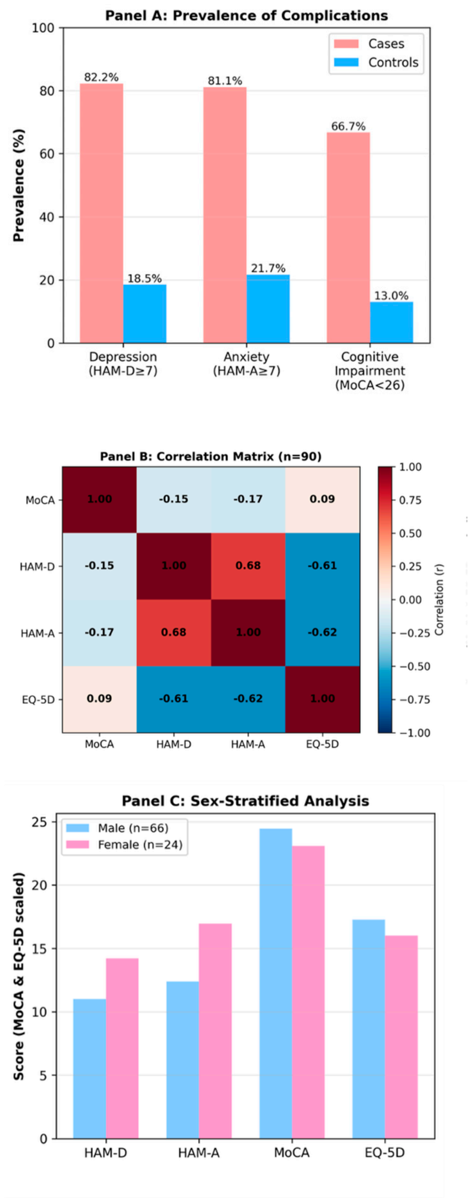

Three-panel summary. Panel A (Prevalence): Cases vs controls—depressive symptoms (HDRS-17 ≥ 7), 82.2% vs 18.5%; anxiety symptoms (HAM-A ≥ 7), 81.1% vs 21.7%; cognitive impairment (MoCA < 26), 66.7% vs 13.0%. Panel B (Correlation heatmap, cases n=90): strong associations between mood and HRQoL (HDRS-17 vs EQ-5D-5L $r = -0.612$***; HAM-A vs EQ-5D-5L $r = -0.625$***), strong HDRS-17–HAM-A correlation ($r = 0.681$***), and negligible MoCA–EQ-5D-5L correlation ($r = 0.092$, NS). The color scale ranges from −1.00 (blue, negative correlations) to +1.00 (red, positive correlations), with neutral colors indicating values close to zero. Diagonal elements represent self-correlations (r = 1.00). Panel C (Sex-stratified outcomes): mean scores by sex among cases.

Supplementary C

STROBE Checklist — Case–Control Study (Completed)

Section/Item	Nº	STROBE recommendation	Where in the manuscript
Title/Abstract	1	(a) Indicate the study design with a commonly used term in the title or abstract; (b) Provide an informative, balanced summary of methods and findings.	Title ("…Case–Control Study"); Abstract (structured IMRaD).
Introduction	2	Background/rationale: explain scientific context and rationale.	Introduction, paragraphs 1–4.
	3	Objectives: state specific aims/prespecified hypotheses.	Introduction, final paragraph (study aims).
Methods	4	Study design: present key elements early.	Methods – Study Design and Participants ("single-center, prospective, observational case–control").
	5	Setting: locations, relevant dates; recruitment and assessment windows.	Methods – Neuroimaging Acquisition (acute MRI; mean 1.3 ± 0.8 days post-event) and Assessments at 90 ± 14 days; recruiting hospital(s).
	6	Participants: eligibility, sources/methods of case ascertainment and control selection; rationale; (b) matching criteria and number of controls per case if applicable.	Methods – Study Design and Participants (18–70 y; TIA/minor stroke NIHSS ≤ 4; DWI-confirmed; age-matched controls; exclusions: prior dementia, pre-morbid mRS > 1, non-Spanish speakers).
	7	Variables: clearly define outcomes, exposures, predictors, potential confounders/effect modifiers; diagnostic criteria.	Methods – Psychopathological, Cognitive, and HRQoL Assessment (HDRS-17, HAM-A, MoCA, EQ-5D-5L; cut-offs); Neuroimaging variables; clinical covariates (age, sex, mRS, social risk).
	8	Data sources/measurement: sources and details of assessment methods; comparability between groups.	Methods – Assessments at 90 ± 14 days (trained, blinded evaluators; standardized protocol); Neuroimaging (1.5 T sequences, blinded neuroradiologists); EQ-5D-5L Spanish value set
	9	Bias: describe efforts to address potential sources of bias.	Methods – Blinding/Training; age-matching for controls; multiplicity control (FDR), HC3 robust SEs, beta-regression sensitivity.
	10	Study size: explain how size was arrived at.	Methods – Regression Modeling (≈10:1 participants:predictors); Limitations (post-hoc power $1-\beta = 0.84$).
	11	Quantitative variables: how handled; groupings and rationale.	Methods – Regression Modeling (z-scoring; binary coding 0/1); cut-offs for HDRS-17, HAM-A, MoCA; EQ-5D-5L utilities.
	12	Statistical methods: (a) all methods incl. confounding control; (b) subgroups/interactions; (c) missing data; (d) matching; (e) sensitivity analyses.	Methods – Statistical Analysis (t/Mann–Whitney/χ^2/Fisher; Cohen's *d*, OR with 95% CI; Pearson/Spearman; hierarchical multiple regression with ΔR^2 and incremental *F*; mediation via PROCESS v3.5, 5,000 BCa; handling of one missing EQ-5D-5L; HC3 & beta-regression sensitivity).
Results	13	Participants: numbers at each stage; reasons for non-participation; consider flow diagram.	Results – Sample Characteristics (N = 182; 90 cases/92 controls; n = 89 in regression due to one EQ-5D missing). Optional flow diagram in Supplement.
	14	Descriptive data: characteristics of participants; exposures/confounders; missing data.	Table 1 (baseline); Results – Sample Characteristics; Table S1 (group contrasts and ORs); note on missing EQ-5D-5L in regression set.
	15	Outcome data: numbers/summaries per group.	Results – Psychopathological, Cognitive, and HRQoL; Figure 1 & Table S1.
	16	Main results: estimates with precision (e.g., 95% CI); adjusted and unadjusted; report category boundaries when categorizing continuous variables.	Results – Regression (Models 1–3; R^2/ΔR^2/*p*; coefficients in Table 3; category thresholds for HDRS-17/HAM-A/MoCA.
	17	Other analyses: subgroups/interactions; sensitivity analyses.	Sex-stratified analysis (Figure S1 Panel C; Table S1 sex block); mediation (Tables S3A–S3B; Figure 3).

Discussion	18	Key results summarized with respect to objectives.	Discussion – Principal Findings.
	19	Limitations: discuss sources of potential bias/precision; direction/magnitude.	Discussion – Limitations (case–control/single time point; measures; unmeasured confounding incl. medications/fatigue; generalizability).
	20	Interpretation: cautious overall interpretation considering objectives, limitations, multiplicity, results from similar studies, and other evidence.	Discussion – Comparison with Prior Work / Mechanisms / Implications.
	21	Generalisability (external validity).	Discussion –
